# Preparation of immunochromatographic strips for rapid detection of early secreted protein ESAT-6 and culture filtrate protein CFP-10 from *Mycobacterium tuberculosis*

**DOI:** 10.1097/MD.0000000000009350

**Published:** 2017-12-22

**Authors:** Xiaoxin Wu, Yeping Wang, Tianhao Weng, Chenyu Hu, Frederick X.C. Wang, Zhigang Wu, Dongshan Yu, Huoquan Lu, Hangping Yao

**Affiliations:** a State Key Laboratory for Diagnosis and Treatment of Infectious Diseases, Collaborative Innovation Center for Diagnosis and Treatment of Infectious Diseases, the First Affiliated Hospital, School of Medicine, Zhejiang University, Hangzhou; b Center of Clinical Experimental Medicine, The People's Hospital of Changxin, Huzhou, China; c Department of Bioengineering, Erik Jonsson School of Engineering and Computer Science, The University of Texas at Dallas, TX.

**Keywords:** colloidal gold immunochromatographic strips, culture filtrate protein 10, early secretory antigenic target 6, *Mycobacterium tuberculosis*

## Abstract

The early secreted protein early secretory antigenic target 6(ESAT-6) and the culture filtrate protein 10 (CFP-10) are 2 antigens that are specific to *Mycobacterium tuberculosis*. These 2 antigens are good targets for tuberculosis (TB) detection.

To rapidly diagnose TB across a variety of samples, we developed colloidal gold immunochromatographic strips (ICSs) based on ESAT-6 and CFP-10.

The strips were evaluated using 233 samples, including sputum, plasma, and pleural effusion samples.

The positive detection rates for ICSs for ESAT-6 and CFP-10 in sputum (culture-positive for *M tuberculosis*) were 100% and 91.2%, respectively. The positive detection rates for ICSs for ESAT-6 and CFP-10 in plasma were 34.1% and 29.4%, respectively. The positive detection rates for ICSs for ESAT-6 and CFP-10 in pleural effusion were 64.7% and 55.9%, respectively. Experimental analysis of culture supernatant showing that the ICS developed for ESAT-6 had a sensitivity of 100% and a specificity of 91.2%. While the ICS developed for CFP-10 had a sensitivity of 91.2% and a specificity of 88.2%.

The validity of the test is limited by source of sample. The technique is sensitive and specific for samples in sputum and culture media but not for plasma or pleural effusion samples. Detection of *M tuberculosis* using ICSs is rapid, simple, and relatively effective; thus, ICSs are a potential screening tool for TB.

## Abstract 2

Supplemental Digital Content is available in the text

## 1 Introduction

The World Health Organization (WHO) estimates that 9.6 million individuals were infected with tuberculosis (TB) worldwide in 2014; approximately 16% of the patients died. Thus, TB is one of the most critical infectious diseases worldwide.^[1–3]^

Conventional microscopy is still widely used to diagnose TB; however, the sensitivity is only 20% to 60%.^[4–6]^ Moreover, 20% of active TB cases, as well as all latent TB cases, cannot be confirmed via traditional methods, including fluorescence microscopy.^[7]^ The WHO recommends liquid culture systems for identifying *Mycobacterium* species and drug susceptibility testing. The BACTEC MGIT 960 system is commonly used.^[8,9]^ However, this is time-consuming and is often unavailable in remote areas without experimental facilities.

Early, rapid, and reliable diagnosis of patients infected with *Mycobacterium tuberculosis (M tuberculosis)* can allow early treatment and prevent further spread of infections; these are key issues in controlling TB. The purified protein derivative (PPD) skin test has been used for a long time, but it yields a high frequency of false-positive results.^[10]^ The enzyme-linked immunospot (ELISPOT) test is a new type of immunological diagnostic tool for TB; this test is much more sensitive and specific than the PPD skin test. However, ELISPOT is time-consuming, laborious, and requires professional staff. The interferon-γ release assay for detecting TB (TB-IGRA) is widely used. This method has relatively high specificity and sensitivity.^[11–13]^ But this method is also time-consuming and laborious. Polymerase chain reaction (PCR) detection is rapid and of high-sensitivity, and soon been widely applied in most TB-endemic countries. However, this method requires complicated and expensive equipment (i.e., a PCR machine) and well trained personnel. Not all communities, especially those in remote areas, can afford application of this testing method.^[14]^

Early secreted antigen 6 (ESAT-6, 6 kDa) and cell filtrate protein 10 (CFP-10, 10 kDa) are 2 specific *M tuberculosis* antigens. Detection of ESAT-6 and CFP-10 are 2 primary proteins that distinguish *M tuberculosis* from Bacillus Calmette Guerin (BCG), promoting the pathogenicity of *M tuberculosis* and allowing the IGRA to be specific for *M tuberculosis*.^[12,13]^ These 2 antigens have been used to rapidly detect TB in blood samples.^[15]^ Previously, we developed an enzyme-linked immunosorbent assay (ELISA) for the detection of ESAT-6 and CFP-10. The ELISA method has a relatively high sensitivity and specificity.^[16]^ However, this method requires 5 to 6 hours as well as specific scientific equipment.

Immunochromatographic strips (ICSs) is a rapid 1-step immunochromatographic assay. All development reagents are incorporated into the analysis strip^[17]^; thus, this is an instant and convenient method. In this study, we developed and evaluated ICSs for the rapid detection of *M tuberculosis* ESAT-6 and culture filtrate protein CFP-10. This assay was found to be rapid, simple, and relatively effective.

## 2 Materials and methods

### 2.1 Materials

ESAT-6 and CFP-10 were prepared and purified in our laboratory. Mouse anti-ESAT-6 monoclonal antibody 10G10 and mouse anti-ESAT-6 monoclonal antibody A8–11 were prepared in our laboratory. Mouse anti-CFP-10 monoclonal antibody immunoglobulin M (IgM) 4D6C9 and mouse anti-CFP-10 monoclonal immunoglobulin G (IgG) 3D4G4 were prepared in our laboratory. Goat anti-mouse second resistance IgG, nitrocellulose (NC) membranes with backing, glass fibers, and absorption pads were purchased from Shanghai Jin Biao Biotechnology (China). Chloroauric acid (gold chloride, AuCl_3_.HCl.4H_2_O) was purchased from Shanghai Chemical Reagent (China). The rest chemicals used in this study were either of analytical purity or of the highest quality available.

### 2.2 Synthesis and characterization of colloidal gold

Colloidal gold particles were prepared according to previously published methods.^[18,19]^ In brief, an aqueous solution of chloroauric acid (50 mL, 0.01% AuCl_3_.HCl.4H_2_O) was refluxed; then, 1.75 mL of 1.0% sodium citrate solution was added. The solution was heated until the color of the solution changed from straw yellow to black to red. Then, the solution was cooled to room temperature and concentrated to 50 mL. The colloidal gold particles were observed using transmission electron microscope and multiskan spectrum microplate spectrophotometer at Zhejiang University.

### 2.3 Optimization and preparation of antibody–colloidal gold conjugates

An antibody solution (monoclonal antibody, 1 mg/mL, 3 μL) was added to 100 μL of the colloidal gold solution (pH 5–9) in triplicate. After 15 minutes, 20 μL of 10% NaCl was added. After 2 hours, the lowest pH that maintained a red color was considered to be the optimum pH. Then, various amount of purified antibody solution (0.1 mg/mL, from 2 μL to 12 μL) were added to wells with 100 μL of colloidal gold solution at the optimum pH. The solutions were incubated at room temperature for 15 minutes. Twenty microliters of 10% NaCl was added and change of color was observed after 2 hours. The minimum amount of antibody required to stabilize the colloidal gold particles was determined. A 20% increase over the minimum amount of antibody was considered to be the optimal amount of protein. Based on the determined conditions, antibody–colloidal gold conjugates were manufactured.

### 2.4 Preparation of ICS

**Figure 1:**
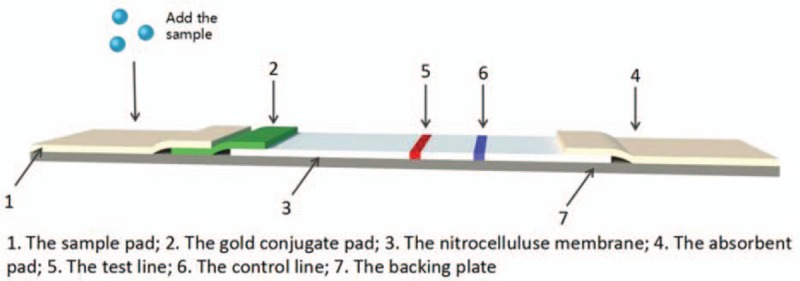
Schematic of immune colloidal gold strip production.

The ICSs were prepared by the method reported by Paek et al.^[20]^ The gold-labeled antibody probe was jetted onto the glass fiber and dried at room temperature. The goat anti-mouse antibody and monoclonal antibody (in phosphate buffer solution) were jetted onto a NC membrane in 2 discrete zones (one for the control experiment). The remaining active sites on the membrane were blocked by incubating with 5% nonfat milk in phosphate buffer solution (PBS) overnight at 4°C. The membrane was washed 3 times with PBS buffer and air-dried. The sample application pad, antibody–gold conjugated pad, NC membrane, and absorption pad were air-dried and laminated. The sample pad had a short overlap (0.2 cm) on the antibody–gold conjugated pad; the antibody–gold conjugated pad and blotting paper overlapped on the NC membrane. This ensured that liquid could be transferred between each layer. A diagram of the immunochromatographic test paper is shown in Figure 1.

### 2.5 Clinical samples

The clinical samples (plasma, pleural effusion, and sputum) were obtained from the sample banks of the Hangzhou Red Cross Hospital and the First Affiliated Hospital of Zhejiang University. The samples were aliquoted and stored at −80°C. The plasma and sputum samples were from 85 pulmonary TB patients treated between January 2012 and January 2016. These 85 patients were diagnosed according to the American Thoracic Society guidelines and Pai et al.^[21–23]^ Patients with both plasma and sputum samples were selected for testing. Of the 85 patients, 38 patients had sputum samples that were cultured positive for *M tuberculosis*; the other 47patients were cultured negative for *M tuberculosis*. Among these 85 patients, 72 were diagnosed with pulmonary TB by the TB-SPOT method. Pleural effusion was also used to validate our ICS method. We selected 34 patients who had TB pleurisy. The pleural effusion samples were obtained from the sample bank. Twenty-eight of the patients were diagnosed by the PCR method. Thirty-one patients were diagnosed by the TB-SPOT method. The remaining 29 patients were not reported to have had TB pleurisy; however, after retesting we found that 3 patients were positive via the PCR method and 2 were positive via the TB-SPOT method. Informed consents were obtained from all the participants. Our ICS was validated and compared with other methods, such as liquid culture method, ELISA, T-SPOT, and PCR. Meanwhile, 38 samples of *M tuberculosis* culture supernatant and 34 samples of non-*M tuberculosis* culture supernatant were provided by our TB diagnostic laboratory; these were used to evaluate the sensitivity and specificity of our ICS method.

### 2.6 Procedure for clinical sample detection

The clinical samples (i.e., pleural effusion, sputum, peripheral blood plasma, or culture supernatant) were applied to the end of the strip, allowing the sample to migrate upward. At least 100 μL was required. If the sputum was too thick, we diluted 1:1 with saline. The liquid was filtered through a 0.4-μm filter. After 10 minutes, an image containing the color signal was produced on the strip, and the results were determined from the color of the test and control lines. If both the detection and control bands turned red, the sample was recorded as positive. If the control band turned red, but the detection band did not, the sample was recorded as negative. If neither band was colored, the test reagents were assumed to be ineffective.

### 2.7 Statistical analysis

All the experiments were conducted by the same person. All the experiments were performed once. The colloidal gold immunochromatographic strip method was compared with ELISA, PCR, and blood TB-SPOT methods using the Pearson *χ*^2^ test. *P* < .05 was considered significant.

## 3 Results

### 3.1 Characterization of colloidal gold particles

All the particle sizes (17.99–20.18 nm) were suitable for the immune colloidal gold test. Different contents of sodium citrate yielded different sizes of colloidal gold particles. Smaller particle size distributions corresponded to more uniform peak widths. The smallest peak occurred with 1.75 mL of sodium citrate; this also yielded uniformly sized colloidal gold particles (Supplementary Figure 1).

### 3.2 Optimization of test paper production conditions

**Figure 2:**
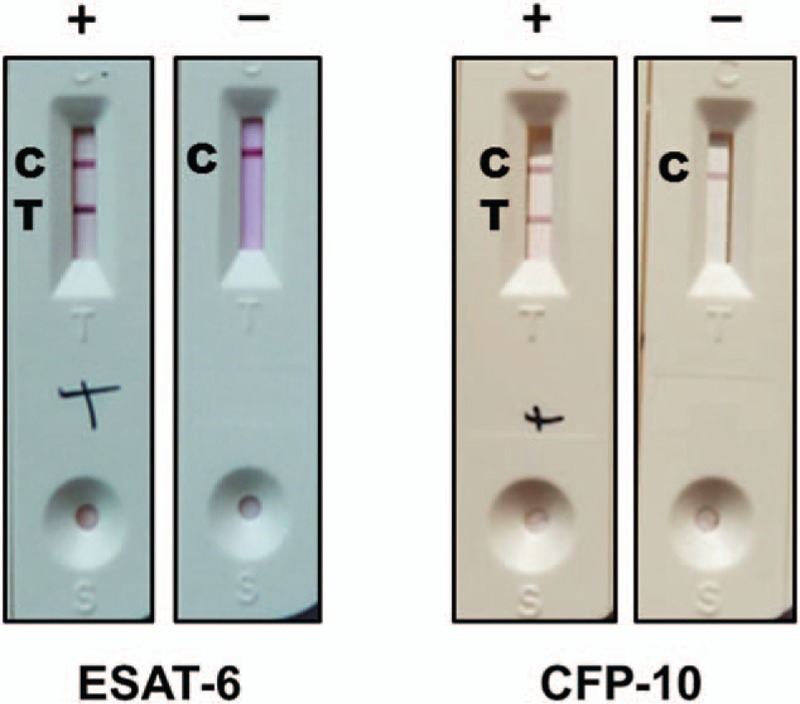
The quality of the colloidal gold immunochromatographic strips. C = control line, CFP-10 = culture filtrate protein 10, ESAT-6 = early secretory antigenic target 6, T = test line.

NC membrane NC140 had the best properties. The relatively hydrophilic polyester fiber membrane VL78 was selected as the colloidal gold pad. A hydrophilic glass fiber film was selected as the sample pad. The optimal preparation of ICSs for ESAT-6 detection were as follows: antibody adsorption pH was 7.0, antibody concentration was 12 μg/mL, sample pad was pretreated with 0.01 mol/L pH 7.4 PBS buffer (with 3.8% borax, 0.5% casein sodium, 1% PVP-10, and 1% Triton X-100), T line concentration was 400 ng/mL, C line concentration was 2.2 μg/mL. With these conditions, the antibody–colloidal gold conjugates had good qualities and the detection sensitivity of the colloidal gold strip was 6.0 ng/mL (Fig. 2).

The optimal preparation of ICSs for detecting CFP-10 were as follows: antibody adsorption pH was 7.5, antibody concentration was 7.2 μg/mL, sample pad was pretreated with 0.01 mol/L pH 7.4 PBS buffer (with 3.5% sucrose and 3.5% BSA), colloidal gold pad was pretreated with 0.01 M pH 7.4 PBS buffer (with 0.25% BSA, 0.25% sucrose, and 0.25% Tween-20), T line concentration was 200 ng/mL, C line concentration was 200 μg/mL. Under these conditions, the antibody–colloidal gold conjugates had good qualities and the detection sensitivity of the colloidal gold strip was 2.4 ng/mL (Fig. 2).

### 3.3 Stability of strip

The stability of the ICSs was investigated at 4°C and at room temperature. The ICSs were tested during 16 seven-day intervals; this showed that the test line was clear. The ICSs remained stable for at least 4 months at room temperature under dry and airtight conditions. The ICS may remain stable for a longer time at 4°C.

### 3.4 Experimental analysis of sputum from TB patients using ICSs and ELISA

Of the 85 sputum samples, 38 were cultured positive for *M tuberculosis*; the remaining 47 were cultured negative for *M tuberculosis*. We first compared the ICS and ELISA methods for detection of sputum that was cultured positive for *M tuberculosis.* The positive detection rate of the ICS for ESAT-6 was 100% (38/38). The positive detection rate of the ICS for CFP-10 was 92.1% (35/38). When using the ELISA method, the positive detection rates for ESAT-6 and CFP-10 were 100% (38/38) and 94.7% (36/38), respectively (Table 1). No statistical differences were observed between the ICS and ELISA methods in the detection of culture-positive sputum samples.

**Table 1:**
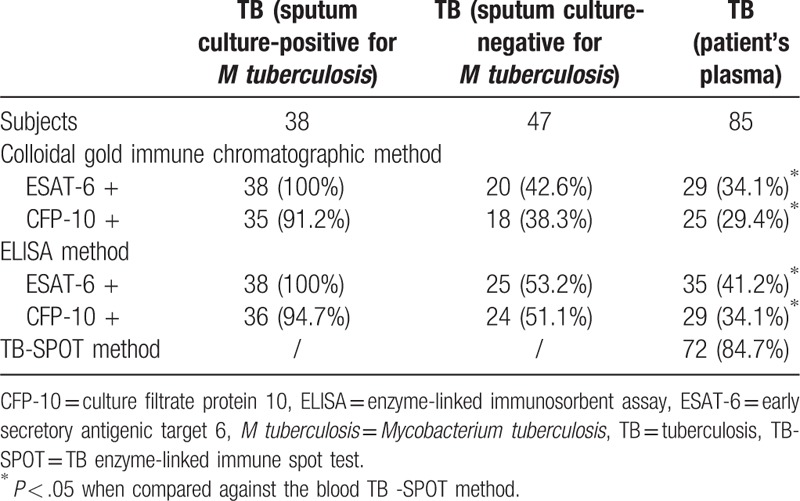
Comparison of the colloidal gold immune chromatographic, ELISA, and TB-SPOT methods for detection of TB in sputum and peripheral blood samples.

Second, we used the ICS and ELISA methods to analyze sputum that was cultured negative for *M tuberculosis.* The positive detection rate of ICS for ESAT-6 was 42.6% (20/47). The positive detection rate of ICS for CFP-10 was 38.3% (18/47).The positive detection rates of ELISA for ESAT-6 and CFP-10 were 53.2% (25/47) and 51.1% (24/47), respectively (Table 1). No statistical differences were observed between the ICS and ELISA methods when detecting sputum that was cultured negative for *M tuberculosis.*

The positive detection rates of ICS for ESAT-6 and CFP-10 in sputum samples from TB patients were 68.2% and 62.4%, respectively. The positive detection rates of ELISA for ESAT-6 and CFP-10 in sputum samples were 74.1% and 70.6%, respectively. There were no statistical differences between the ICS and ELISA methods.

### 3.5 Experimental analysis of plasma from the TB patients using ICSs and ELISAs

Of 85 TB patients, 72 patients were diagnosed with pulmonary TB based on positive TB-SPOT results. We also tested these samples using ICSs and ELISAs. The positive detection rate using ICSs for ESAT-6 was 34.1% (29/85). The positive detection rate using ICS for CFP-10 was 29.4% (25/85). The positive detection rates using ELISAs for ESAT-6 and CFP-10 were 41.2% (35/85) and 34.1% (29/85), respectively (Table 1). There were no significant differences between the ICS and ELISA methods; however, TB-SPOT was more sensitive than both the ICS and ELISA methods (*P* < .001) (Table 1).

### 3.6 Experimental analysis of pleural effusion using ICSs and ELISA

Of the 34 pleural effusion samples, 28 tested positive via PCR and 31 tested positive via TB-SPOT. The positive detection rate using ICS for ESAT-6 was 64.7% (22/34). The positive detection rate using ICS for CFP-10 was 55.9% (19/34). The positive detection rates using ELISA for ESAT-6 and CFP-10 were 73.5% (25/34) and 67.6% (23/34), respectively. There were no significant differences between the ICS and ELISA methods for pleural effusion samples (Table 2). The positive detection rates using the PCR and TB-SPOT methods were 82.4% (28/34) and 91.2% (31/34), respectively. Both the TB-SPOT and PCR methods were more sensitive than the ICS method for pleural effusion samples (ICS for ESAT-6 vs .TB-SPOT: *P* = .008; ICS for CFP-10 vs. TB-SPOT: *P* = .001; ICS for CFP-10 vs. PCR: *P* = .018).

**Table 2:**
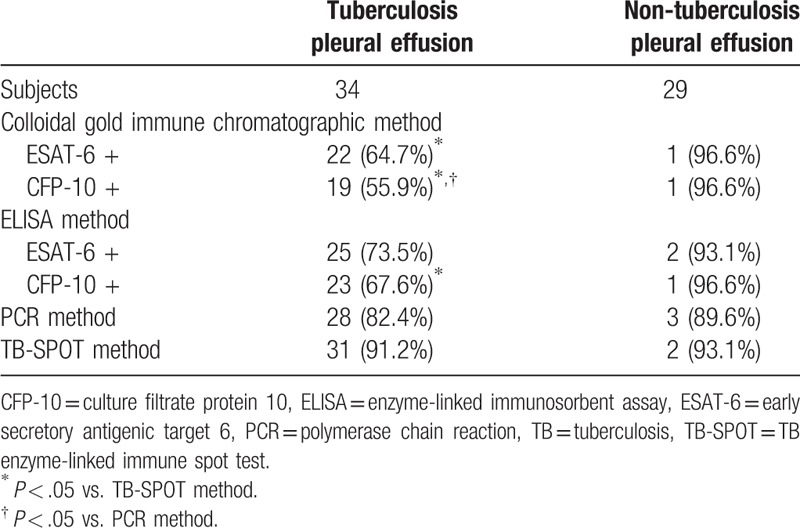
Comparison of the colloidal gold immune chromatographic, ELISA, PCR, and TB-SPOT methods for detection of TB in pleural effusion samples.

Patients tested using ICS included 29 without TB pleurisy, 3 patients positive for *M tuberculosis* using the PCR method, and 2 patients tested positive for *M tuberculosis* using the TB-SPOT method. The false-positive rate using ICS was 3.4% for both ESAT-6 and CFP-10. The false-positive rates using ELISA were 6.9% for ESAT-6 and 3.4% for CFP-10. There were no significant differences in false-positive rates between the ICS, ELISA, PCR, and TB-SPOT methods (Table 2).

### 3.7 Experimental analysis of culture supernatant using ICSs

Culture supernatants from 38 *M tuberculosis* samples and 34 non-*M tuberculosis* samples were used to evaluate the sensitivity and specificity of our ICS method. The ICS developed for ESAT-6 had a sensitivity of 100% and a specificity of 91.2%. The ICS developed for CFP-10 had a sensitivity of 91.2% and a specificity of 88.2% (Table 3).

**Table 3:**
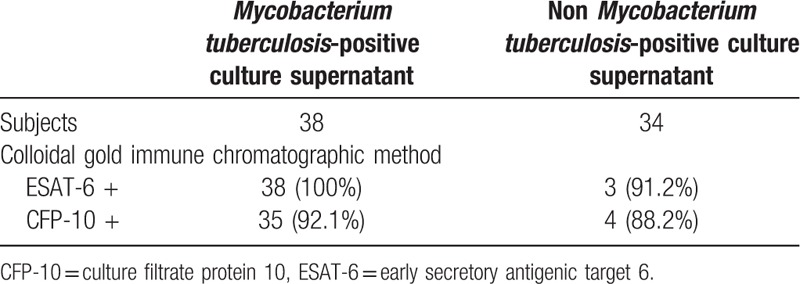
Results of the colloidal gold immune chromatographic method for detecting culture supernatant samples.

## 4 Discussion

TB is a major public health problem. In the past decade, the emergence of diverse drug-resistant TB strains, the prevalence of human immunodeficiency virus leading to co-infection with *M tuberculosis*, the flow of high-risk groups or immigrants, and gaps in TB control in many countries has led to the global reoccurrence of TB. This has attracted the attention of the international community.^[24–26]^ The WHO has marked TB as one of the most potent infectious diseases, and estimates there are almost 2 billion people infected with TB worldwide; of these, 20 million are estimated to be TB patients. There are 8 million new cases of TB each year, and 2 million people die from TB every year. Among these, only a few received a regular laboratory diagnosis.

Early diagnosis is crucial to preventing the spread of TB. The PPD test is commonly used clinically, but there are many restrictions of its use. It cannot distinguish between different types of TB infections and has a high frequency of false-positives. The smear method was previously used to identify *M tuberculosis*, but the positive rate was too low. The separation culture method is the criterion standard for the diagnosis of TB; however, it takes nearly 1 month to complete. Thus, this method is not conducive to early diagnosis. TB-IGRA can distinguish between BCG and TB. It is widely used with high specificity and sensitivity,^[11–13]^ but the testing process is relatively complex and time-consuming. The PCR and TB-SPOT methods are highly sensitive and specific. These 2 methods are ideal for TB detection, but these methods require specialized equipment. Furthermore, the testing process is complex and the cost is relatively high. These 2 methods may be good choices for developed medical institutions, but they could not be widely applied in remote areas that lack the necessary equipment and well trained personnel. Since most TB cases occur in remote and/or poor areas, there is an urgent need to develop a quick and easy diagnosis method with a relatively high specificity and sensitivity.

The colloidal gold immunochromatographic test has been widely used as a rapid diagnostic test for various diseases, including *Toxoplasma gondii*, malaria, chlamydia, mycoplasma, paragonimiasis, and schistosomiasis.^[27–29]^ An ICS was prepared and evaluated for the rapid detection of *M tuberculosis*. We also compared the ICS method with the ELISA method, which was previously developed to detect TB.^[17,30]^

There was no significant difference in the positive detection rate between the ICS and ELISA methods. It should, however, be noted that the ICS does not require specialized equipment or trained operators and provides easily readable results in only 15 minutes. The cost of each ICS is about 3 US dollars. The positive detection rates of the ICS method for ESAT-6 and CFP-10 in sputum samples (culture-positive for *M tuberculosis*) were 100% and 91.2%, respectively; this is adequate for early screening. For culture negative sputum, our ICSs showed nearly 40% positive results, which was comparable to the ELISA results. There are many reasons that could cause a negative culture result. Many of the patients were treated with antibiotics before sampling, which may restrain the growth of TB bacterium; sample quality varied; and the quantity of TB bacterium in sputum was important for the culture. Based on the considerations mentioned above, we believed that our methods could mainly detect the presence of TB bacterium that did not grow out in the laboratory.

In our study, we also tested our ICS on culture supernatant, pleural effusion and plasma. Experimental analysis of culture supernatant showed that this ICS for ESAT-6 and CFP-10 had a sensitivity of 100% and 91.2%, respectively. Whereas the positive detection rates of the ICS method for ESAT-6 and CFP-10 in pleural effusion samples were 64.7% and 55.9%, respectively. The positive detection rate of the ICS method in plasma samples was about 30%. The validity of the test was limited by source of sample. Meanwhile, our study also has disadvantage of limited sample size used to verify the ICS method, which may also contribute to error.

In general, the ICS method is a promising technology for screening TB that can be commercially manufactured. This will be helpful to screening suspected patients in endemic areas, providing an alternative diagnoses method in primary hospitals, and assessing treatment efficacy. In the future, we will continue to evaluate the effect of our ICS method by expanding the sample size.

## 5 Conclusion

We developed an ICS for the rapid detection of ESAT-6 and CFP-10. This ICS can be effectively used to diagnose TB in sputum by providing easily readable results in 15 minutes. Our method can be commercially manufactured, and widely utilized to screen suspected patients.

## Acknowledgments

The authors thank their colleagues for their help with collecting materials and samples in the sample bank.

## Supplementary Material

Supplemental Digital Content
