# Supplementary material for: Preparation of immunochromatographic strips for rapid detection of early secreted protein ESAT-6 and culture filtrate protein CFP-10 from Mycobacterium tuberculosis
Source: Medicine (Baltimore). 2017 Dec 22;96(51):e9350. doi: 10.1097/MD.0000000000009350 (PMC5758221; doi:10.1097/MD.0000000000009350)

**Supplementary figure 1** The absorption spectrum diagram under different addition of citrate solution. Note: Different volume of 1.0% sodium citrate solution was added to chloroauric acid (50 mL, 0.01% AuCl_3_.HCl.4H_2_O) . The absorption spectrum diagram was detected under different addition of citrate solution


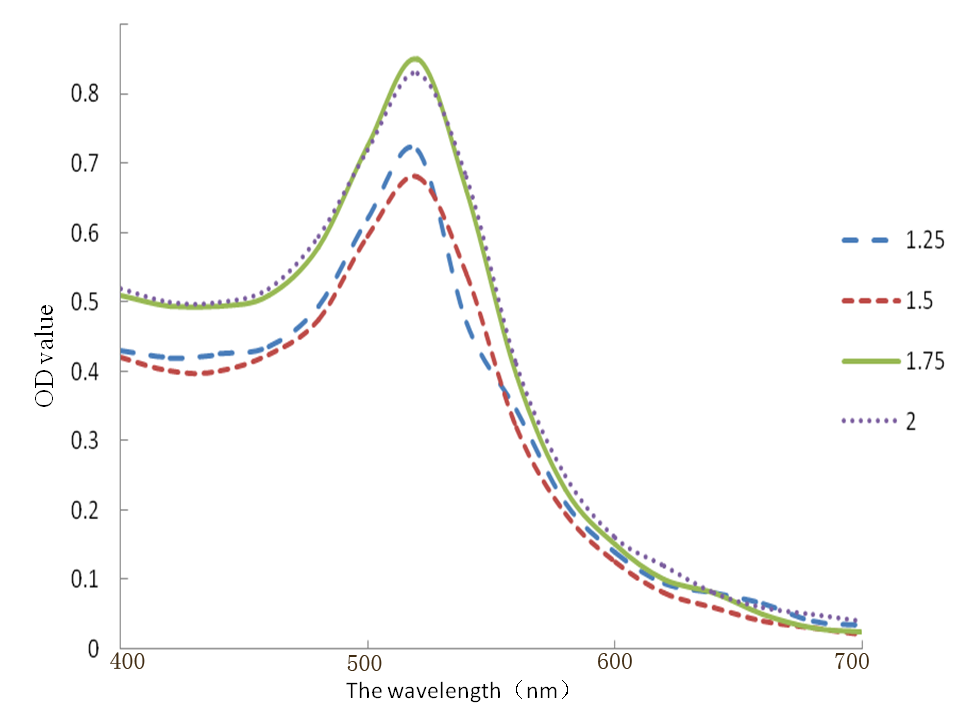

Supplement: Supplemental Digital Content [file medi-96-e9350-s001.doc]
